# The Wilhelm Bernhard workshop: half a century of collegiality

**DOI:** 10.1080/19491034.2019.1649844

**Published:** 2019-08-12

**Authors:** Thoru Pederson

**Affiliations:** Department of Biochemistry and Molecular Pharmacology, University of Massachusetts Medical School, Worecster, MA, USA

In this issue, we are pleased to have a report of the 26^th^ Wilhelm Bernhard Workshop, held May 20–24, 2019 in Dijon, France. Without going into the higher mathematics (in which the cumulative years do not define the anniversary number), 2019 marked the 50^th^ year since this biennial conference was launched. I was not there at the founding but can reach back far enough to offer some historical perspectives and, most emphatically, applaud the cell biologist whose name adorns this conference.

The science of cell structure arose mainly in Germany, in the theatre of pathology. Key landmarks were the invention of phase-contrast microscopy by Frits Zernike (Nobel Prize in Physics, 1953) and pioneering work by Ernst Ruska (Nobel Prize in Physics, 1968) using electron beams instead of photons. Wilhelm Bernhard (born in a small Swiss village in 1920 and having ground his own telescope lenses a a boy, perhaps foreshadowing his career in microscopy) came up after these discoveries and like so many scientists of his time and place whom we would today retroactively label as ‘cytologists’ or ‘cell biologists’, he had come through the portal of pathology. A man of boundless energy and exceptional intellectual penetration, after obtaining his medical degree he trained with the cancer pathologist Charles Oberling at Villejuif, France. Oberling was interested in cancer viruses and asked Bernhard to set up an electron microscopy laboratory to hunt for them. (This is likely what set in motion one of Bernhard’s major lifelong interests, the cytological detection of ribonucleoprotein). His earliest contributions were the development of cryoprotectants in electron microscopy, as well as the introduction of hydrophilic embedment materials to facilitate the subsequent action of various enzymes to digest suspected molecular components. His development of the so-called retrogressive EDTA-uranyl acetate method brought him particular recognition in the nucleus field due to its ability to selectively stain RNA-containing particles at the EM level. This innovation together with his avid interest in ribonucleoprotein (*vide supra*) naturally led him to the nucleolus, as well as to the inter-chromatin granule clusters (later becoming known as ‘nuclear speckles’).

Wilhelm Bernhard’s research achievements brought him many high prizes but this is not the place to go into his career into any greater depth than I have just done. Readers wishing to know more are referred to two evocative memoirs published shortly after his death [1,2], his youthful 58 years making this a great tragedy.

Most American scientists of my generation vividly recall meeting European scientists of the previous generation and how amazed we were by their knowledge of history, art, literature, and music and while my examples on this phenotype were cell biologists like Christian de Duve and George Palade, the record of everyone who knew him is that Wilhelm Bernhard had the same level of vast erudition. One American cell biologist who knew him well told me that Wilhelm loved Schubert and always encouraged colleagues to admire in particular the *Über Liebe* songs of this composer. He also was a devotee of Marcel Proust and it has been said that his home had a Proustian ambience with old books and antiquities. It is thought that this was his motivation to always include a concert and/or cultural excursion at each meeting. But the image of Wilhelm sitting in an armchair and listening to a phonograph or reading Goethe is not the complete picture. Something also stirred him and led him to passionately operate beyond the confines of his laboratory or the comfort of his living room. I do not think any of us today can know from where this sprang but it remains as his signature in the field of cell biology. There was a sense that he acutely perceived the East-West split underway.

Wilhelm Bernard got the idea, in 1966 or so, that cell biology was too cloistered in particular ‘schools’, typically defined by imperious leaders with rigid ideas and with little ear for younger scientists around them. In this respect Wilhelm Bernhard was an anarchist, in the finest mold, sensing that there was a systemic defect. He was of course also a citizen of the 20^th^ century in Europe, and while from a non-bellicose country, he certainly felt the angst of how science in Europe had been imperiled by the rise of fascism.

Bernhard corralled a small number of colleagues and rallied them around the idea of a small meeting, but an international one. Those two defining ideas today sound like a total oxymoron but in 1966–1968 when his idea was brewing it was a radical concept. He further argued that the meeting should be held at different places each time (here again one senses his strong phobia about a ‘center’) and that the meetings should emphasize an egalitarian principle, in which everyone would present their work. A corollary doctrine, which was vividly evident when I first began attending, was that no country in the extended family of the Wilhelm Bernhard Workshop was to be regarded as anything other than trying its best, after whatever top science in that country had been destroyed and was now in recovery. I was very moved by witnessing this, as were all my American colleagues in attendance. One suspects this too Wilhelm anticipated as a way the Workshop could benefit the entire community.

**Figure 1.**
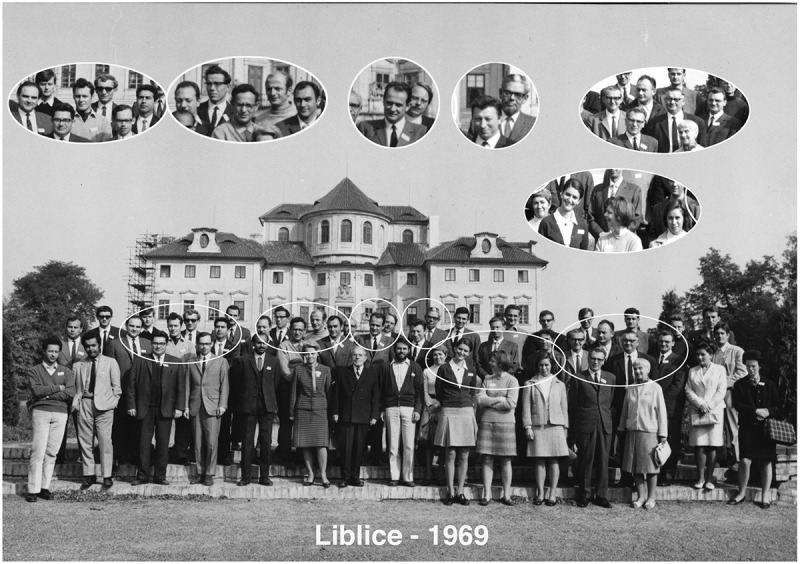
Participants at the first Workshop. Wilhelm Bernhard is in the foreground of the center blow-up.

The first conference was held in 1969 (Figure 1). It was initially called ‘The Nucleolar Workshop’, shortly became ‘The International Workshop on the Cell Nucleus’, and in recent years ‘The Wilhelm Bernhard Workshop’. (This later name seems just as it honors him without dilution, the field of this major meeting now widely known.) The meetings have been held at a diversity of sites (Table 1) and there has been a striking degree of commitment and consensus by the International Committee over all the years. Some of us who serve on this committee have done so for 25–30 years or more and while this might not be the standard model of turnover, we simply love the Wilhelm Bernhard Workshop too much. As the more senior of us members say ‘We will have to be carried out on a stretcher.’ Wilhelm would have been pleased to see this commitment. He honored our field by launching this meeting. We are reciprocally honored to serve this mission. He would also be pleased to know that, at least in my experience, there has never been dissent about membership on the International Committee, the siting of a planned meeting or the design of the program. The planning, and the meetings themselves, have been the most apolitical activities in most of our careers and this too would make Wilhelm happy.

**Table 1.** Sites of the Wilhelm Bernhard workshops.

1969 Liblice, Czechoslovakia	1995 Spa, Belgium
1971 Lake Balaton, Hungary	1998 Lac-Delage, Canada
1973 Abisko, Sweden	1999 Prague, Czech Republic
1975 Varna, Bulgaria	2001 Arcachon, France
1977 Salamanca, Spain	2003 Pavia, Italy
1979 Weimar, Germany	2005 Münsterschwarzach Abbey, Germany
1981 Safed, Israel	2007 St. Andrews, Scotland
1983 Banyuls-sur-Mer, France	2009 Ustron, Poland
1985 Krakow, Poland	2011 Riga, Latvia
1987 Stevensbeek, The Netherlands	2013 Debrecen, Hungary
1989 Suzdal, USSR	2015 Vienna, Austria
1991 Les Diablerets, Switzerland	2017 Nizhny Novgorod, Russia
1993 Lake Balaton, Hungary	2019 Dijon, France

Wilhelm Bernhard was anything but an elitist so when the idea arose after his tragic death in 1978, that a Medal in his name should be conferred at the Workshop, there was a concern that this might not resonate with his wishes. But it was felt that a Medal would serve to perpetuate his name and contributions and, with him unable to cast a dissenting vote from on high, the motion passed. The medal has been awarded to those (Table 2) who have been deemed to have made important research contributions in the field of the nucleus, with the awarding committee each time also taking into consideration the degree to which the candidate has operated in the ‘manner of Wilhelm’ as to the values of international collegiality that he exemplified. The range of international participation in the Workshop has, of course, expanded over the years, as the field of the molecular and cell biology of the nucleus itself expanded in many countries, and also as certain political barriers that prevailed in 1969 eased in the subsequent decades. Many of the nations represented in 2019 did not even exist, as sovereign entities, in 1969 (Table 3).

**Table 2.** Recipients of the Wilhelm Bernhard medal.

1987 Oscar J. Miller	2005 Bertil Daneholt
1989 Harris Busch	2007 Joseph G. Gall
1991 Werner W. Franke	2009 William T. Garrard
1993 Masami Muramatsu	2011 Klaus Scherrer
1995 Karel Smetana	2013 Tom Misteli
1999 Thoru Pederson	2015 Marion and Thomas Cremer
2001 Eliza Izaurralde	2017 Gisele Bonne
2003 Stan Fakan	2019 Ronald Hancock

**Table 3.** Nationalities represented.

1969	2019
Belgium	Austria
Canada	Canada
Czechoslovakia	China
France	Czech Republic
Germany	Estonia
Great Britain	France
Hungary	Georgia
Italy	Germany
Netherlands	Hungary
Sweden	Italy
Switzerland	Japan
United States	Latvia
	Poland
	Russia
	Senegal
	Ukraine
	United States

There is one more observation that is worth mentioning. In all the many Wilhelm Bernhard Workshops I attended, I was always struck by the fact that for the students it was not only their first meeting, often the case, but was their first international meeting, almost always the case. When some of us recall the first meeting we attended as students was some jumbo thing, where we felt dilute, insecure and unworthy, the presence of students in an engaged and active participatory role at the small, intimate Wilhelm Bernhard Workshops over all the years is yet another jewel in its crown. For them, this first meeting has taught how collegial and international science can be. In this way, Wilhelm’s prescient idea also continues to radiate and inspire.
